# Transbrachial Catheter-Assisted Thrombolysis and Bailout Stenting for Massive Postoperative Pulmonary Embolism

**DOI:** 10.1016/j.jaccas.2025.104156

**Published:** 2025-07-23

**Authors:** Safia Ouarrak, Tomas Cieza, Guylaine Gleeton, Andres Rhul, Paola Ulacia, Jocelyn Gregoire, Zoltan Ruzsa, Olivier F. Bertrand

**Affiliations:** a Quebec Heart-Lung Institute, Quebec, Quebec, Canada; b Invasive Cardiology Division, University of Szeged, Szeged, Hungary

**Keywords:** intravascular ultrasound, pulmonary circulation, stents

## Abstract

**Background:**

A 61-year-old patient developed acute dyspnea and hypoxemia 72 hours after open lung lobectomy. An urgent computed tomography angiography revealed an acute pulmonary embolism with complete left pulmonary artery occlusion.

**Case Summary:**

The patient was referred for urgent catheterization by a multidisciplinary team due to bleeding risks. Using right transbrachial access, the left pulmonary artery was recanalized. After balloon angioplasty, intravascular imaging guided the optimal placement of a stent to resolve vessel recoil. Local low-dose thrombolysis was initiated after partial reopening. After 24 hours, control angiography showed significant improvement in pulmonary artery perfusion.

**Discussion:**

To our knowledge, this is the first reported case of using a coronary stent with local thrombolysis via brachial access to treat acute pulmonary embolism. The procedure successfully restored vessel patency with no complications. This case emphasizes the potential of catheter-based interventions, especially when intravenous thrombolysis is contraindicated.

**Take-Home Message:**

Innovative catheter-based interventions with stenting and local thrombolysis via transbrachial approach may offer a valuable option for managing acute pulmonary embolism in select patients.

## Graphical Abstract

**Figure:**
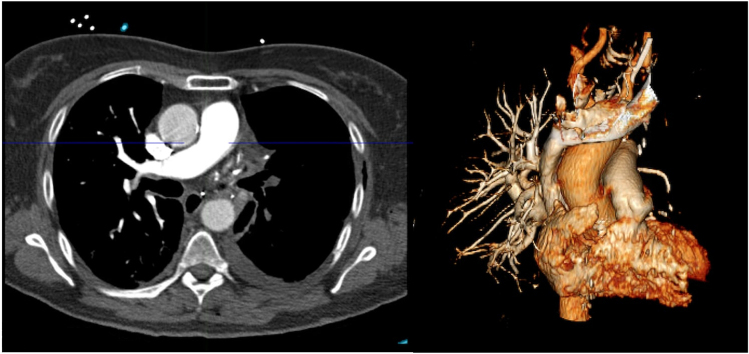

## History of Presentation

A 61-year-old woman presented with severe dyspnea and refractory hypoxemia 72 hours after undergoing a left upper lobectomy with a double sleeve procedure for a recurrent adenocarcinoma in the left upper lobe. On examination, the patient was apyretic. She was tachypneic, with a respiratory rate of 30 breaths/min. Her blood pressure was 100/60 mm Hg. Oxygen saturation remained at 88%, despite the use of supplemental oxygen. She also developed rapid atrial fibrillation at 180 beats/min.

**Take-Home Messages**

• The use of a transbrachial approach for local thrombolysis in acute PE helped to minimize access site–related bleeding risks.
• This case highlights the importance of a personalized approach in managing acute PE, where balloon and stent angioplasty with intravascular imaging guidance can provide an alternative when classical treatments are contraindicated or insufficient.

## Past Medical History

The patient had a history of hypertension and underwent a right upper lobectomy for adenocarcinoma in 2005. She had presented with a recurrence of adenocarcinoma in the left upper lobe, leading to a left upper lobectomy with a double sleeve procedure.

## Differential Diagnosis

Given the patient's dyspnea and the immediate postoperative course, the differential diagnosis included cardiac arrhythmia, such as rapid atrial fibrillation, heart failure, postoperative hemorrhage, pulmonary embolism (PE), pneumonia, and acute respiratory distress syndrome.

## Investigations

**Figure 1:**
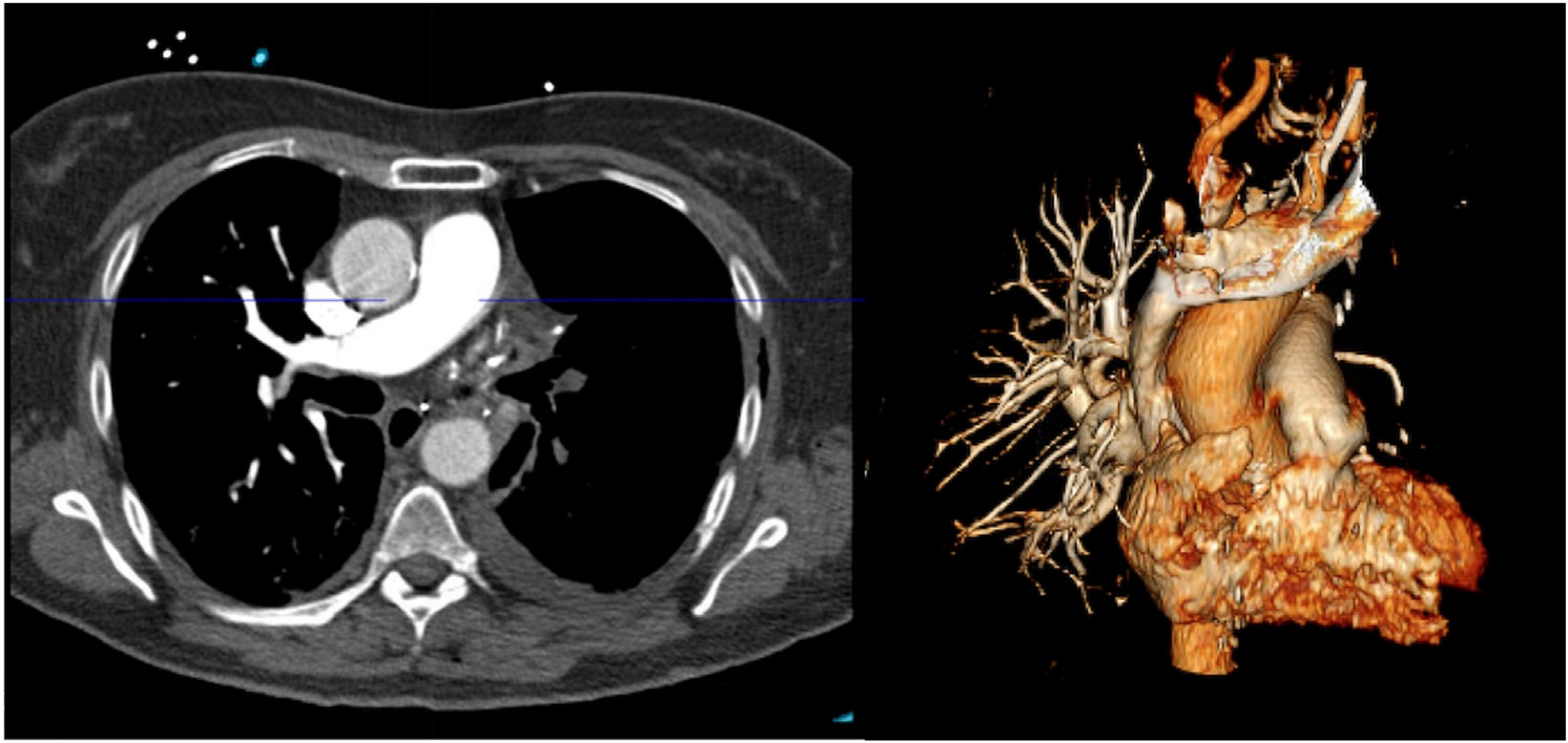
Thoracic Computed Tomography Angiography Revealing Acute Left Pulmonary Embolism

Electrocardiogram showed a rapid atrial fibrillation with T-wave inversions in the right precordial and inferior leads. An emergency thoracic computed tomography (CT) angiography revealed a massive PE with acute complete occlusion of left pulmonary artery (Figure 1). Transthoracic echocardiogram showed normal left ventricular function, without dysfunction or dilatation of the right ventricle; pulmonary pressures could not be estimated from tricuspid regurgitation due to technical reasons and poor echocardiographic echogenicity. The hemogram was normal, and cardiac troponins were elevated (48 ng/L; normal range: <14 ng/L).

## Management

**Figure 2:**
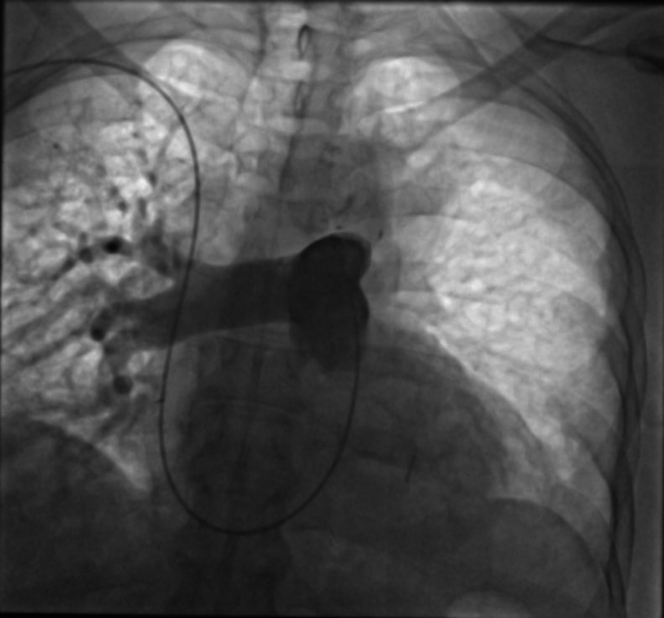
Pulmonary Angiography Confirmed the Proximal Total Occlusion of the Left Pulmonary Artery

**Figure 3:**
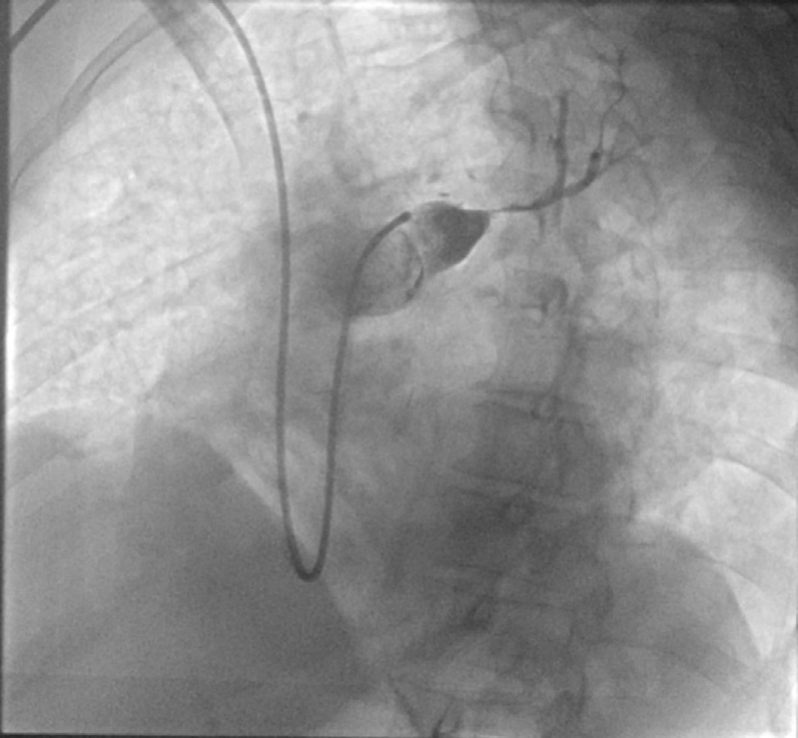
Pulmonary Angiogram Showing the First Result of the Initial Recanalization After Balloon Angioplasty

**Figure 4:**
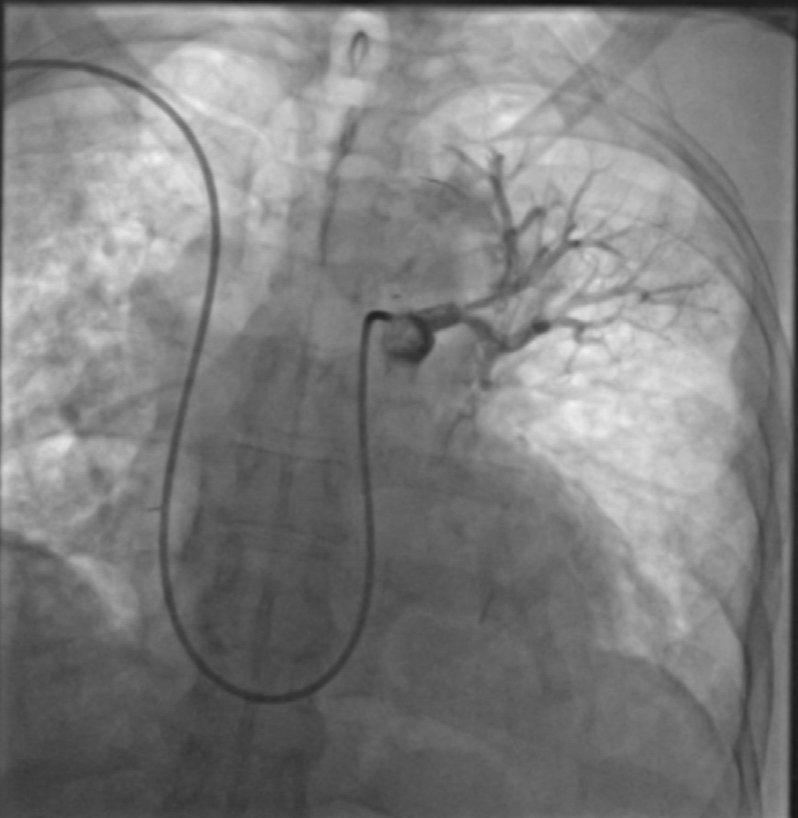
Pulmonary Angiogram Demonstrating the Outcome After Stent Deployment, With Unsuccessful Recanalization of the Inferior Branch

**Figure 5:**
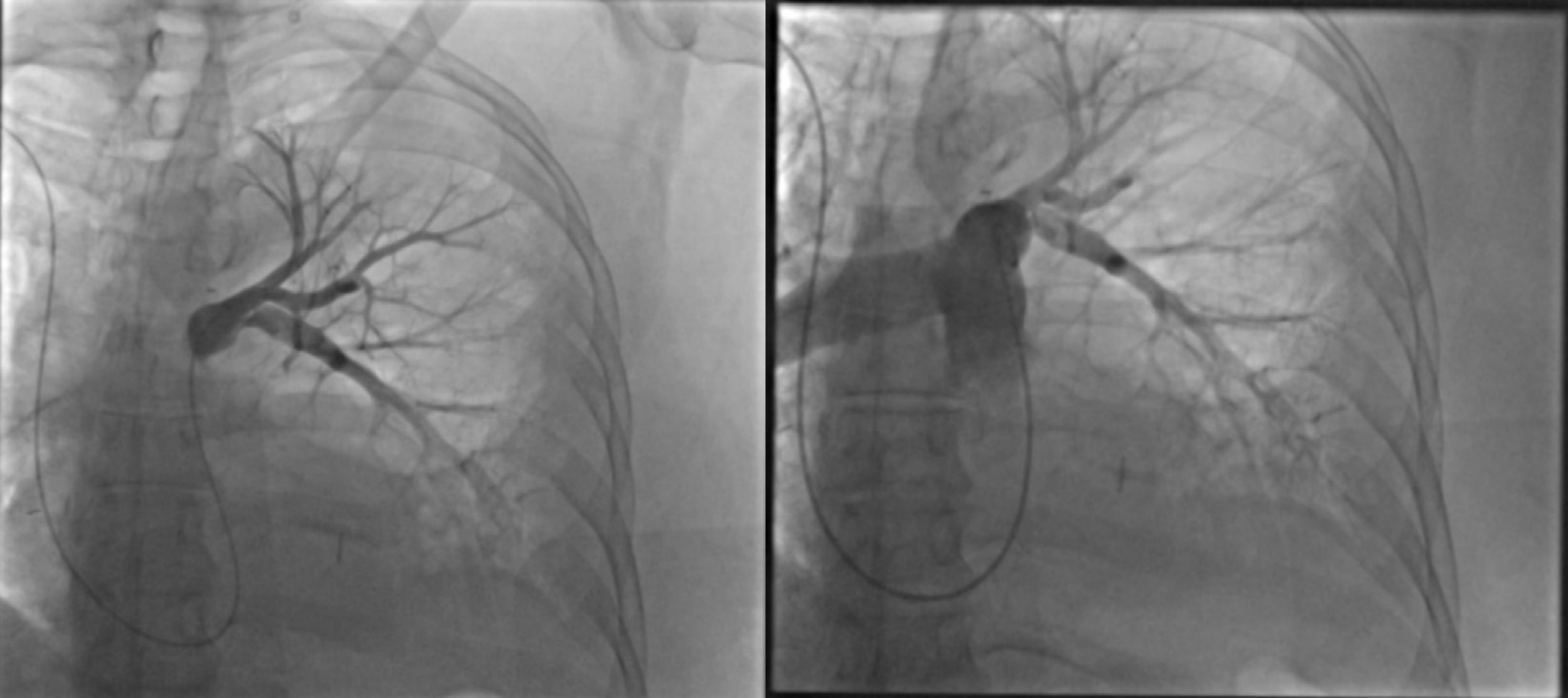
Pulmonary Angiography Control 24 Hours After Local Thrombolysis Showing Successful Reperfusion of the Basal Trunk and Revascularization of the Remaining Branches of the Left Lower Lobe

The patient received a high-flow nasal oxygenation and a continuous unfractionated heparin infusion. After multidisciplinary evaluation, the patient was referred for right heart catheterization and intervention. A 7-F Slender sheath (Terumo Medical) was inserted via a puncture of the humeral vein to obtain vascular access. An initial angiography was performed using a pigtail catheter; pulmonary angiography confirmed a total occlusion in the proximal segment (Figure 2). Initial recanalization was attempted using 0.035 Terumo Glidewire Advantage navigated through a 7-F multipurpose catheter for the superior branch, followed by a 0.014 guidewire for the inferior branch (Balance HeavyWeight, Abott), and sequential balloon angioplasties first with a 2.0- × 30-mm balloon at 18 atm for 60 seconds, then with a 3.5- × 30-mm balloon at 17 atm for 120 seconds (Figure 3). Mechanical aspirations through the 7-F guiding catheter did not retrieve significant blood clots. Despite multiple attempts with balloon angioplasty, no significant recanalization and perfusion could be achieved. Intravascular ultrasound (IVUS) revealed a large thrombus burden and vessel recoil in the proximal left pulmonary artery (Video 1). Further balloon angioplasties with 4.5-mm and 5.5-mm balloons were performed. To definitively address the vessel recoil and impending vessel occlusion, a 5.0- × 13-mm bare-metal stent was deployed in the ostium of the left pulmonary artery and further inflated with a 6-mm balloon at 22 atm, yielding optimal results without signs of thrombosis, dissection, or stent malapposition on postprocedural intravascular imaging (Video 2). However, recanalization of the distal inferior branch was unsuccessful (Figure 4). A local thrombolysis protocol was then initiated with a 50-mg alteplase infusion over 24 hours through a 4-F catheter placed distal to the stent and secured externally. Then 24 hours after local thrombolysis, follow-up angiography revealed successful reperfusion of the basal trunk with partial revascularization of the lower lobe branches; however, irregularities suggested residual thrombi. Thrombolysis was extended for an additional 24 hours, but the patient developed hypotension and mild respiratory deterioration revealing a hemothorax confirmed by a thoracoabdominal CT without any hemorrhage elsewhere. Local thrombolysis was discontinued and followed 48 hours later by intravenous unfractionated heparin. Postintervention, the patient demonstrated significant clinical improvement. Pulmonary angiography control performed 24 hours after angioplasty confirmed successful revascularization of the left pulmonary artery, with restored blood flow to the basal trunk and its branches (Figure 5).

## Discussion

PE is one of the most severe complications after thoracic surgery such as lobectomy. Mortality rates in acute massive PE ranges from 3% to 15%,^1^ with 30,000 to 50,000 PE hospitalizations resulting in death annually in the United States.^2^ The European Society of Cardiology guidelines have stratified PE risk into high, intermediate (further stratified into intermediate-high and intermediate-low), and low risk.^3^ The application of these guidelines in this case was limited due to the specific nature of the patient's condition. We considered that our clinical case did not represent a classic PE; therefore, we thought that the current recommendations for the management of acute PE might not have been applicable to our case. We hypothesized that this was an in situ thrombosis mechanism in the pulmonary artery for the following reasons: the homolateral location of the PE suggested a local mechanism. Additionally, the nature of the intervention—a double sleeve procedure aimed at reconstructing the pulmonary artery—may have contributed to its injury and potential thrombosis. The patient did not present any personal thromboembolic risk factors, such as a history of thrombophilia, hormone therapy, or previous venous thromboembolic disease. For these reasons, and based on multidisciplinary assessment, we considered the patient at risk for rapid clinical deterioration due to the massive appearance of the PE on the CT pulmonary angiography, combined with her history of right lobectomy. We also considered intravenous thrombolysis, but it carried a significant bleeding risk, particularly in the postoperative course.

Indeed, the primary goal of PE treatment is to reduce mortality by reopening the pulmonary artery and restoring blood flow. Traditional treatments include intravenous anticoagulation with unfractionated heparin and thrombolytic therapies with several agents and modified protocols regarding doses and timing of delivery. However, these methods are often ineffective or contraindicated in the postoperative period due to the high risk of surgical bleeding.

Our clinical case illustrates this situation where we faced a patient at high risk of hemorrhage. Therefore, it was decided to proceed with an interventional approach. In our center, the radial approach is the default access site for coronary intervention, and brachial access is used for right heart catheterization. Hence, we opted for right brachial access using thin-walled radial sheaths. Brachial access offers several advantages over femoral access in interventional cardiology and radiology procedures. A study focused on local thrombolysis performed via the femoral access reported a complication rate of 7.5%.^4^ Percutaneous intervention including thrombus aspiration, balloon angioplasty, stent implantation, and IVUS guidance could be performed uneventfully. Furthermore, the 4-F catheter could be left in place to administer local thrombolysis and secured externally without complications on the local access site. In our case, we initially opted for intravascular imaging to better understand the mechanism of pulmonary artery occlusion. We observed a large thrombus completely obstructing the vascular lumen, which allowed us to perform initial predilatation with balloons to reopen the lumen. However, our balloon angioplasty alone proved insufficient, which was not surprising given the high thrombotic burden. Similar to coronary treatment, the absence of flow and evidence of vessel recoil after balloon dilation justified the choice of stenting because it helps ensure vessel patency. To our knowledge, there are no published data on stenting or balloon angioplasty for acute PE, making this the first reported case. Our treatment was guided by IVUS to size the stent and verify its proper apposition. In this case, we used stent-assisted recanalization as a novel approach to treat a totally occluded pulmonary artery. The literature on stenting for acute PE is poor, with most studies focusing on stenting the pulmonary artery in cases of chronic pulmonary stenosis, where the aim is to improve blood flow. A study using a sheep model of PE demonstrated the technical feasibility of temporarily placing and removing a newly designed pulmonary stent to recanalize located embolic occlusions in severe PE.^5^ The experimental evaluation showed rapid and significant improvement in circulatory parameters after stent placement.

The final IVUS control after stent deployment showed good stent apposition without local complications postangioplasty. However, the angiographic result was partial because the distal inferior branch remained nonpatent. We then opted for in situ thrombolysis to dissolve the residual thrombus and optimize the outcome. Catheter-directed thrombolysis (CDT) is a minimally invasive reperfusion option for acute PE. This technique administers the thrombolytic drug directly to the pulmonary artery thrombus, achieving the highest drug concentrations which may accelerate clot dissolution. This targeted delivery can also be more effective at dissolving the thrombus because it bypasses the body's natural reflex to divert pulmonary blood from obstructed arteries, precisely where the thrombolytic agent is most required.^6^ As a result, CDT uses much lower doses of thrombolytic agents than systemic infusions (10%-20% of the systemic dose), potentially reducing the risk of hemorrhagic side effects.^7^ A randomized pilot study compared CDT with standard anticoagulation in patients with intermediate- to high-risk acute PE. CDT showed superior improvement in right ventricular function, pulmonary pressure reduction, and thrombus burden compared with standard therapy, without causing life-threatening bleeding.^8^ The findings suggest CDT can be a safe and effective treatment for acute PE.^8^

In our case, we administered 50 mg of alteplase via infusion over 24 hours. The optimal doses of alteplase for CDT are not yet fully established in the literature. The SEATTLE II (A Prospective, Single-arm, Multi-center Trial of EkoSonic® Endovascular System and Activase for Treatment of Acute Pulmonary Embolism) study, a prospective, single-arm, multicenter trial, used 24 mg of alteplase (1 mg/h for 24 hours) over pharmacomechanical catheter–directed therapy, which combined fibrinolytic therapy with ultrasound’s mechanical disruption of the thrombus.^9^ Similarly, the OPTALYSE (A Randomized Trial of the Optimum Duration of Acoustic Pulse Thrombolysis Procedure in Acute Intermediate-Risk Pulmonary Embolism) trial used a lower dose of alteplase (4-12 mg per lung).^10^ In these studies, the delivery system was mechanical, enhancing the effect of the fibrinolytic agent. Additionally, all patients in both studies received intravenous heparin at a full therapeutic dose. In our case, we chose to administer one-half of the conventional dose rather than the lower dose reported in the literature because we used a standard catheter for delivery. Additionally, the patient was not on intravenous heparin due to the postoperative context, which posed a high bleeding risk, along with dual antiplatelet therapy after recent stent deployment.

## Follow-Up

**Figure 6:**
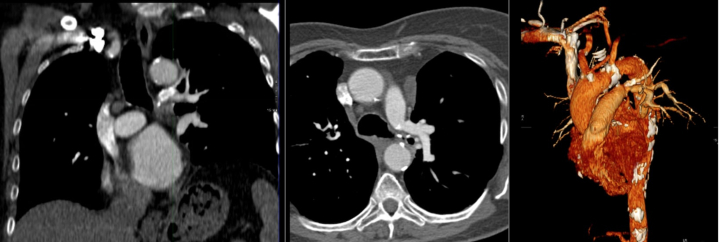
Follow-Up Thoracic Computed Tomography Angiography Showing Stent Patency 7 Years After the Acute Episode

The patient was discharged after 12 days of hospitalization with oral anticoagulation therapy. There was no evidence of pulmonary hypertension or PE recurrence at CT pulmonary controls. The follow-up is currently being conducted 7 years after the last hospitalization. Results were excellent, with stent patency and no signs of pulmonary hypertension (Figure 6). The management of dual antiplatelet therapy was also worth discussing. Our patient was discharged on aspirin and low-molecular-weight heparin which was pursued for 6 months for the duration of chemotherapy.

This clinical case could open new therapeutic avenues for managing a postlung surgery PE associated with a high hemorrhagic risk.

## Conclusions

This case underscores the importance of individualized treatment strategies in managing acute PE in the perioperative course. It highlights the potential of stenting as a viable alternative when conventional treatments are contraindicated or insufficient. As the first documented case of stenting for acute PE, this report opens new avenues for innovation in interventional cardiology and critical care, with the potential to improve patient outcomes in similar high-risk scenarios.

## Funding Support and Author Disclosures

The authors have reported that they have no relationships relevant to the contents of this paper to disclose.

## Appendix

Video 1 Intravascular Ultrasound Showing Significant Thrombus Burden and Vessel Recoil in the Proximal Left Pulmonary Artery

Video 2 Intravascular Ultrasound Showing Optimal Results, With No Signs of Thrombosis, Dissection, or Stent Malapposition
